# Vibrating Mesh and Jet Nebulizer Performance in Pediatric Respiratory Support: A Multi-Modality In Vitro Comparison

**DOI:** 10.3390/pharmaceutics18050575

**Published:** 2026-05-06

**Authors:** Ronan MacLoughlin, Ann-Marie Crowe, Michael Scully, Brendan D. Higgins

**Affiliations:** 1 Emerging Technologies, Aerogen Ltd., IDA Business Park, Dangan, H91 HE94 Galway, Ireland; rmacloughlin@aerogen.com; 2 School of Pharmacy and Biomolecular Sciences, Royal College of Surgeons, D02 YN77 Dublin, Ireland; 3 School of Pharmacy and Pharmaceutical Sciences, Trinity College, D02 PN40 Dublin, Ireland; 4 Pediatric Intensive Care Unit, Assistance Publique des Hôpitaux de Paris, Hôpital Necker Enfants Malades, Université Paris Cité, 75015 Paris, France; ann-marie.crowe@aphp.fr; 5 Discipline of Anaesthesia, School of Medicine, Clinical Sciences Institute, University of Galway, H91 YR71 Galway, Ireland; michael.e.scully@universityofgalway.ie; 6 Discipline of Physiology, School of Pharmacy and Medical Sciences, Human Biology Building, University of Galway, H91 W5P7 Galway, Ireland

**Keywords:** aerosol, blow-by, HFNT, jet nebulizer, mechanical ventilation, pediatric, vibrating mesh nebulizer

## Abstract

**Background:** The aim of this study was to assess in vitro nebulized drug delivery during invasive and non-invasive ventilation, comparing jet nebulizers (JN) and vibrating mesh nebulizers (VMN) across various pediatric ventilation models. **Methods:** Drug delivery performance was compared between a continuous output JN (Aquineb) and VMN (Aerogen Solo A-VMN). The non-invasive model simulated a spontaneously breathing 9-month-old child using an anatomically correct upper airway model and breathing simulator. The invasive model used a mechanical ventilator with heated humidifier in a pediatric breathing circuit with an endotracheal tube. Nebulizers were driven with supplemental oxygen at manufacturer-recommended rates and positioned at approved locations. Absolute inhaled dose, delivery rate and residual volume were assessed using face mask, mechanical ventilation, high-flow nasal therapy and blow-by delivery methods. Dose was quantified using spectrophotometric analysis. **Results:** During spontaneous breathing, A-VMN delivered almost double the dose of the evaluated JN (*p* < 0.001), with a significantly faster delivery rate (*p* < 0.001) and lower residual volume (*p* < 0.0001). During mechanical ventilation, A-VMN demonstrated a greater than 3-fold increase in delivered dose (*p* < 0.0001) and faster delivery (*p* < 0.0001), with reduced residual volume (*p* < 0.001). During high-flow nasal therapy, delivery via nasal cannula was affected by gas flow rate for both devices, with A-VMN consistently delivering greater doses. A-VMN delivered significantly greater salbutamol doses during blow-by delivery. **Conclusions:** VMN demonstrated significantly superior dose delivery, faster delivery rates and reduced residual volumes compared to the evaluated JN across all tested pediatric respiratory support modalities. These in vitro findings provide important performance data for evidence-based device selection and warrant clinical investigation to determine potential therapeutic benefits in pediatric populations requiring aerosol therapy during respiratory support.

## 1. Introduction

Acute wheezy episodes are a common presentation in pediatric populations, often necessitating nebulized medication. Many children with severe asthma have substantial morbidity, and require ‘back-to-back’ nebulizers, while some may need critical care admission for further therapeutics and possibly invasive ventilation. Asthma is the most common chronic respiratory condition affecting approximately 13.5% of children [1]. It is characterized by periods of reversible airflow obstruction caused by bronchoconstriction of hypersensitive airways. Exacerbations clinically manifest as wheezing, coughing, chest tightness and shortness of breath. Apart from asthma, acute wheeze can occur in other conditions such as viral bronchitis, respiratory allergies and anaphylaxis. Such conditions vary in severity from mild to life-threatening events requiring hospital admission and emergency treatment [2]. Additionally, aerosolized pediatric drug delivery is associated with lower delivery efficiencies and higher dose variability which may contribute to inconsistent clinical outcome [3,4].

A wide variety of devices such as pressurized metered dose inhalers (pMDIs) and nebulizers are utilized to aerosolize drugs, and the interactions between these devices and lung delivery is the subject of considerable research interest [5]. Conventional jet nebulizers (breath-actuated, breath-enhanced or constant-output) are the most commonly used nebulizers in hospitals, primarily due to their competitive cost and prior staff experience, with drug delivery known to vary significantly when using this type of nebulizer [6,7]. Inhaled short-acting β2 agonists are first-line agents in pediatric asthma patients and have proven effectiveness [1,8,9,10]. Should initial therapies fail and bronchospasm persist, critical care admission may be needed. A large percentage of patients admitted to hospital with acute severe asthma are admitted to intensive care units, with many of those admitted being placed on high-flow nasal therapy (HFNT) or invasive mechanical ventilation (MV), which is associated with greater morbidity and mortality [11,12].

Overall clinical outcome and increased healthcare costs could be altered by improved drug delivery in the initial treatment phase and during mechanical ventilation, if initiated [13]. While pMDIs and spacers have been shown to be at least as effective as jet nebulizers at treating mild and moderate asthma attacks in hospitalized patients [14], there are limited data on which to base recommendations for device selection in severe, life-threatening asthma in pediatric populations [15]. Aerosols produced by vibrating mesh nebulizers have shown potential in providing greater inhaled and pulmonary deposition compared with jet nebulizers during HFNT and MV in both in vitro and animal models [16,17,18], and surveys of clinical practice suggests that as many as 75% of pediatric cases are prescribed aerosolized medications during HFNT, with VMN used as often as 77% of the time and usually integrated into the HFNT circuit [19]. Furthermore, incorporating a nebulizer system into HFNT circuits has been shown to improve comfort in infants with bronchiolitis, as well as offer the potential for continuous applied pressure [20], without the interruption caused by breaking the circuit during drug refill of a JN [21].

A recent survey of aerosol therapy practices in Spanish Pediatric Intensive Care Units revealed significant variability in clinical practice, with notable inconsistencies in device selection, circuit positioning and delivery parameters across different respiratory support modalities (HFNC, NIV and MV). While vibrating mesh nebulizers were the most commonly used devices (91.7%), their placement within ventilator circuits frequently diverged from evidence-based recommendations. All respondents unanimously agreed on the urgent need for pediatric-specific consensus guidelines to standardize aerosol therapy delivery in critically ill children [22].

Previous studies have compared the performance of JNs and VMNs; however, a significant gap exists for comprehensive evaluations across multiple pediatric delivery modalities using a standardized methodology. This study addresses this gap by providing direct performance comparisons across common clinical scenarios in pediatric respiratory care, including face masks, mechanical ventilation, high-flow nasal therapy and blow-by methods. Such comparative performance data are essential for evidence-based device selection and may inform optimization strategies for aerosol therapy in this vulnerable population, potentially reducing the need for escalation to invasive respiratory support.

## 2. Materials and Methods

### 2.1. Nebulizers

Two commercially available nebulizer systems were assessed. A JN (Aquineb, AFP Medical Ltd., Rugby, UK) and an A-VMN (Aerogen^®^ Solo nebulizer, in combination with Aerogen Ultra chamber and Aerogen USB controller, Aerogen Ltd., Galway, Ireland) were used. The JN was operated at 6 L·min^−1^ as per the manufacturer’s recommendations. A-VMN does not require a driving gas flow for operation; however, a supplemental gas flow of 2 L·min^−1^ was applied when connected to the Ultra chamber, representing likely clinical use. The droplet size, reported as volumetric mean diameter (VMD), was characterized as previously described [23] and measured as 3.39 ± 0.87 μm and 4.47 ± 0.09 μm for the JN and A-VMN respectively.

### 2.2. Mechanical Ventilation

**Figure 1:**
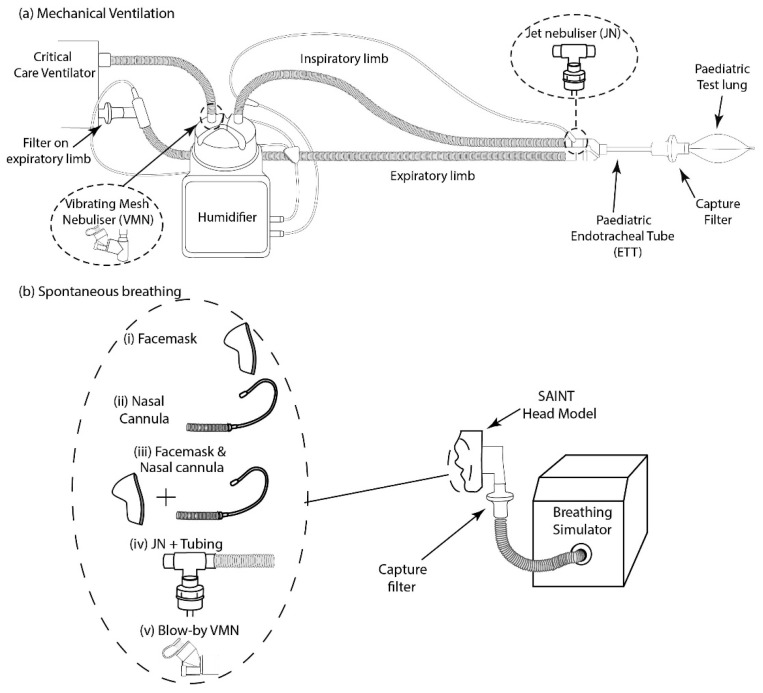
Experimental setup. (**a**) Mechanical ventilation model: the JN was positioned between the inspiratory limb and patient wye; the A-VMN was positioned at the inlet of the heated humidifier (dry side). (**b**) Spontaneous breathing model: for face mask delivery, the nebulizer was positioned immediately proximal to the mask inlet; for high-flow nasal therapy, the A-VMN was integrated into the Airvo™2 circuit via the Aerogen Ultra chamber proximal to the nasal cannula, and the JN was applied via face mask placed over the nasal cannula; for blow-by delivery, both devices were positioned 6 cm from the face of the airway model.

In line with previous reports [18,20], mechanical ventilation was simulated using a ventilator (Servo-i, Maquet, Rastatt, Germany), a heated humidifier (MR850, Fisher and Paykel, Auckland, New Zealand) and a pediatric breathing circuit (RT200, Fisher and Paykel) with an endotracheal tube (ETT, 5 mm ID, Flexicare Medical Ltd., Mountain Ash, UK) terminating in a pediatric-appropriate lung (1 L (#2810000), InterSurgical^®^, Dublin, Ireland) (Figure 1). Aerosol delivered to the distal end of the ETT, i.e., lung dose, was captured on a collection filter (303EU, Vyaire Medical, Dublin, Ireland) placed between the ETT and lung. The JN was placed between the inspiratory limb and patient wye, and the A-VMN was placed at the inlet of the heated humidifier (dry side), in accordance with manufacturer guidance and published recommendations for each device type [24,25] (Figure 1a). An expiratory filter was placed between the circuit and ventilator in order to prevent both ventilator contamination and emissions to the environment during test [26]. The breathing parameters used are provided in Table 1 below.

### 2.3. Spontaneous Breathing Model

Aerosol drug delivery was assessed for face mask, nasal cannula and the blow-by method during simulated spontaneous breathing. The Sophia Anatomical Infant Nose–Throat (SAINT) model, a nose only upper airway stereolithographic model reconstructed from the CT scan data of a 9-month-old infant, was used [28]. A breathing simulator (ASL 5000, Ingmar Medical, PA, USA) was used to generate patient breath in line with international standards [29].

The pediatric face masks supplied by the nebulizer manufacturers were used and a good fit against the model face was ensured to minimize leaks and consequent aerosol losses to the environment. The nebulizer was positioned immediately proximal to the face mask inlet in both cases (Figure 1b). The JN was supplied with a non-valved open aerosol face mask; the VMN was supplied with a valved face mask. High-flow nasal therapy (HFNT) was applied using the Airvo™2 system, with A-VMN nebulizer adapter (Fisher and Paykel, Auckland, New Zealand). This nebulizer adapter was designed by both Fisher and Paykel and Aerogen, for use with the Aerogen Solo A-VMN™ vibrating mesh nebulizer [30]. A-VMN performance was assessed with aerosol delivered via the Airvo™2 junior nasal cannula (OPT316, Fisher and Paykel).

JNs are contraindicated for use when connected to HFNT patient circuits [21,31]. This is due to the interference with the applied therapeutic oxygen concentrations and gas flow rates [32]; therefore JN performance during concurrent HFNT was assessed in this modality using a JN aerosol mask placed over the nasal cannula [22]. This reflects clinical practice and is in line with previous reports [30,33,34]. For both nebulizer types, HFNT was applied at 2 L·min^−1^ and 11 L·min^−1^ via the nasal cannula [30,34,35].

The blow-by method was assessed by connecting the JN to corrugated tubing, with the end of the tubing placed 6 cm away from the face. The tubing was used to direct the aerosol towards the face. The blow-by method using the VMN consisted of the A-VMN held on its side, 6 cm from the face. There was no requirement for tubing when utilizing the A-VMN (Figure 1b).

### 2.4. Aerosol Dose Characterization

For each test, the nebulizer was included in the patient circuit as described above. A nominal dose of 2.0 mL of 2.0 mg/mL of salbutamol (Salamol Steri-Neb™, Teva Pharmaceuticals, Dublin, Ireland) was pipetted into the nebulizer being tested and nebulized until end of dose. End of dose was considered to be nebulization until sputter plus 1 min for the JN, and to the end of visual aerosol generation for the A-VMN. The aerosol dose delivered to the distal end of the ETT, and at the tracheal level of the head model, was captured on a collection filter. The mass of drug on the filter was quantified using UV spectrophotometry at 276 nm and interpolation on a standard curve of salbutamol concentrations. Results are expressed below as a percentage of the nominal dose placed in the nebulizer. The rate of nebulization was determined by recording the time taken to nebulize the dose. Residual volume was measured by gravimetric analysis using a precision scale.

### 2.5. Statistical Analysis

Data are expressed as mean ± SD. Statistical analysis was carried out by using unpaired *t*-tests for comparison between nebulizers (GraphPad Prism version 10.1.1). Statistical significance was set a priori at *p* value of less than 0.05. No data were recorded excluded. All data were recorded in triplicate (*n* = 3).

## 3. Results

### Aerosol Drug Mass Delivery

The drug dose, expressed as drug mass delivered to the distal end of the ETT during mechanical ventilation, or the level of the trachea during spontaneous breathing, is presented in Table 2 below.

**Figure 2:**
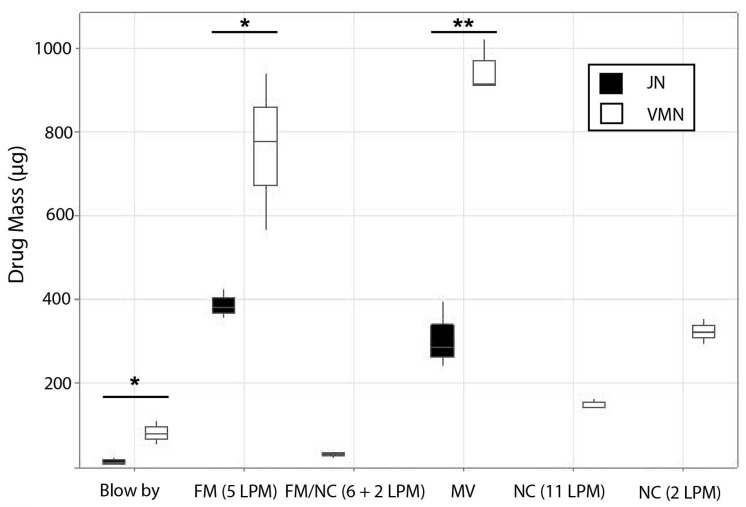
Lung model deposition from nebulizers used in different face mask and ventilator configurations. FM: face mask; MV: mechanical ventilation; NC: nasal cannula; LPM: liters per minute. * *p* < 0.05; ** *p* < 0.01.

**Figure 3:**
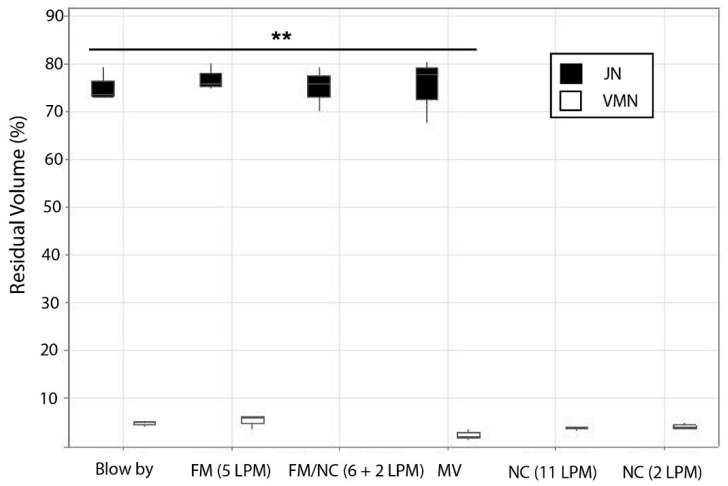
Residual medication volumes from nebulizers used in different face mask and ventilator configurations. FM: face mask; MV: mechanical ventilation; NC: nasal cannula; LPM: liters per minute. ** *p* < 0.01.

During mechanical ventilation, A-VMN delivered a greater than 3-fold dose of salbutamol compared with JN in this pediatric model (*p* = 0.00041) (Figure 2). The % nominal tracheal dose for A-VMN was 23.75 ± 1.60% compared to 7.70 ± 2.01% for JN. In a model of a spontaneously breathing pediatric patient, the A-VMN with face mask delivered a 1.96-fold greater amount of salbutamol than JN under normal breathing conditions (*p* = 0.027) (Figure 2). This equates to a delivery efficiency as measured by tracheal dose (19.07 ± 4.68% versus 9.71 ± 0.89% respectively). In testing, nasal cannula plus A-VMN at one of two different flow rates (FiO_2_ 1.0 at 2 L or 11 L·min^−1^) delivered 324.51 ± 30.89 μg or 150.00 ± 12.82 μg of salbutamol, respectively. The % nominal tracheal doses for VMN aerosol were 8.11 ± 0.78% (2 L·min^−1^) and 3.75 ± 0.32% (11 L·min^−1^), resulting in residual volumes of 4.16 ± 0.67% and 3.75 ± 0.44%, respectively, for the two flow rates as shown in Figure 3.

When a face mask was used in combination with nasal cannulas to deliver JN-produced aerosol, the drug dose delivered was 30.39 ± 6.12 μg, which equates to a % tracheal dose of 0.76 ± 0.15%. This delivery mode resulted in a JN residual volume of 74.94 ± 4.64%. And finally, when comparing JN and A-VMN directly using the blow-by method of delivery, a 6-fold increase in the mass of drug was found (Table 2) (*p* = 0.01567), with a highly significant lower residual volume in the A-VMN also observed (*p* = 0.00001).

During spontaneous breathing, the rate of delivery by A-VMN was faster compared to JN for the normal breathing parameters tested (211.86 ± 57.15 μg·min^−1^ vs. 119.36 ± 14.79 μg·min^−1^ respectively, although not statistically significant (*p* = 0.0533)). During mechanical ventilation in the model tested, the A-VMN delivered salbutamol at a significantly faster rate than the JN (281.71 ± 0.64 μg·min^−1^ vs. 116.34 ± 36.05 μg·min^−1^ respectively; *p* = 0.00135). Aerosol delivery via high-flow nasal cannulas by A-VMN at one of two different flow rates (2 or 11 L·min^−1^) delivered 88.94 ± 8.84 μg·min^−1^ and 46.34 ± 4.03 μg·min^−1^ of salbutamol respectively. While comparing the rate of delivery for JN (3.90 ± 2.31 μg·min^−1^) and A-VMN (23.92 ± 7.72 μg·min^−1^) directly using the blow-by method of delivery, a 6-fold increase in the rate of delivery was found (*p* = 0.0126).

## 4. Discussion

This study demonstrated that A-VMN increased both dose and rate of drug delivery compared to JN across all pediatric models tested. Residual volume was significantly lower with VMN in both the spontaneous breathing and mechanical ventilation models. This is a system-level comparison: each device was evaluated with its respective manufacturer-recommended interface, accessories and supplemental gas flow, as used in clinical practice. Performance differences reflect the entire delivery system rather than nebulizer hardware alone.

### 4.1. Delivery Efficiency

The results of this study are in broad agreement with other studies comparing JN and VMN delivery efficiency under all conditions tested and may be a function of how the aerosol is generated, the percent emitted as a respirable aerosol and the amount of drug remaining in the nebulizer [36,37,38].

The superior delivery efficiency observed with A-VMN reflects fundamental differences in aerosol generation mechanisms, with VMNs using piezoelectric technology to vibrate a precisely engineered mesh at ultrasonic frequencies, producing more consistent aerosol output compared to jet nebulizers that exploit gas-driven mechanisms for aerosol generation [39]. In contrast, jet nebulizers use a high-velocity gas stream directed through a liquid reservoir to generate droplets using the Bernoulli effect, with baffles removing larger particles; this process produces inherently variable aerosol output dependent on operating conditions [5,6]. The 3-fold higher dose delivery observed in both spontaneous breathing and mechanical ventilation models demonstrates the clinical significance of device selection in pediatric aerosol therapy. Recent pediatric emergency department experience confirms that these efficiency advantages translate to meaningful clinical outcomes, with A-VMN use associated with 36 min shorter time to disposition and 15.8 mg lower total albuterol dose per patient compared to traditional jet nebulizers [40].

It is notable that the JN used produced a smaller VMD (3.39 µm) than the A-VMN (4.47 µm), a difference that theoretically favors deeper airway penetration for this JN in terms of aerodynamic conditions. The consistently superior delivered dose observed with the A-VMN likely reflects output efficiency and near-complete drug discharge rather than a particle size advantage, and the magnitude of the A-VMN benefit should be interpreted in this context. These performance advantages may translate to clinical benefits in acute care settings. The consistent aerosol output and lower residual v olumes mean that pediatric patients receive more predictable dosing with less variability between treatments, reducing the risk of treatment failure and unnecessary escalation to more invasive respiratory support.

### 4.2. Nebulizer Position During Mechanical Ventilation

Nebulizer placement within the ventilator circuit significantly affects delivery efficiency during mechanical ventilation, with changes in position altering delivery by 35–61% depending on device type and respiratory support mode [24]. In pediatric ventilator models, positioning nebulizers proximal to the humidifier rather than near the Y-piece has been shown to increase aerosol delivery across multiple device types. Berlinski and Willis (2015) demonstrated that continuous-output jet nebulizers positioned proximal to the humidifier increased delivery 2–3 fold compared to Y-piece placement [25]. For conventional continuous VMN, humidifier inlet placement has been shown to perform best in pediatric models [41,42]. Proximal placement also provides consistent nebulizer orientation and reduced device weight [43]. In the present study, manufacturer guidelines dictated device placement: the JN was positioned before the Y-piece while the A-VMN was placed proximal to the humidifier. While device positioning can influence delivery efficiency [25], the magnitude of the difference observed between devices suggests circuit position alone does not account for the A-VMN performance advantage.

### 4.3. Patient Interface

Standard non-valved open aerosol face masks reduce drug delivery by allowing aerosol to escape to the atmosphere, with emissions onto the face and into the eyes also reported. Environmental emission patterns vary significantly between nebulizer types and patient interfaces [44,45,46]. The recommended valved face mask used in conjunction with the A-VMN, in combination with low supplemental gas flow, has previously been demonstrated to reduce this wastage, thereby facilitating greater delivery efficiency in pediatric in vitro models [47].

Previous studies have demonstrated advantages when an A-VMN, used in conjunction with a proprietary valved holding chamber (Aerogen Ultra), is compared to constant-output JN with a corrugated tube in delivering aerosol in vitro [45] and to patients [47]. The opening of the expiratory mouthpiece valve on expiration facilitates aerosol bolus build-up in time for the next inhalation and an expiratory filter can be attached to capture exhaled aerosol, though this was not applied in the present study.

Artificial airways such as endotracheal tubes (ETTs) significantly influence aerosol deposition during mechanical ventilation, with delivery efficiency inversely related to ETT internal diameter [4,20,48]. This is especially critical in pediatric patients where ETT inner diameters can be as small as 2.5 mm in premature infants and 3–5 mm in infants and young children [4,49]. Small diameter ETTs create substantial aerosol losses through increased turbulence and impaction [48], with infant ETTs being 30–50% narrower than the tracheal diameter [49], resulting in significant depositional losses. In addition to affecting flow patterns, aerosols traveling through pediatric ventilator circuits experience size reduction to less than 2 μm, which may affect the site of deposition within the respiratory tract [24]. Recent in vitro pediatric testing has demonstrated that VMN positioning between the Y-piece and ETT, combined with optimized particle size, can achieve delivery efficiencies up to 4.5% of nominal dose in 9-month-old models with a 5 mm ETT, though this remains substantially lower than non-invasive delivery routes [20]. The smooth interior surfaces of ETTs may facilitate laminar flow compared to upper airways [48], potentially offsetting some losses when aerosol characteristics are optimized. However, the paucity of in vivo data in mechanically ventilated pediatric patients represents a critical knowledge gap requiring clinical validation [4,24].

### 4.4. Residual Volume

This study demonstrated a marked advantage in output efficiency, reflected in the negligible residual drug volume in the A-VMN reservoir following nebulization in agreement with previously published studies [34,50,51]. The residual volume remaining in the VMN reservoir at the end of nebulization in both spontaneous breathing and mechanical ventilation was significantly lower compared to the JN. The enhanced drug utilization efficiency with VMNs (>1–3% residual versus 34–46% for JNs) represents a critical advantage in pediatric care, where weight-based dosing precision is paramount [38,52]. This improved efficiency may reduce the need for repeated nebulizer treatments commonly employed during acute exacerbations, while reducing medication costs and minimizing exposure to preservatives in respiratory solutions that can cause cumulative bronchoconstriction [53,54].

These residual volume advantages are particularly relevant in pediatric populations, where smaller tidal volumes, narrower airways and less consistent breathing patterns already compromise aerosol delivery efficiency. The 2–3-fold dose increase with A-VMN may partially offset these inherent physiological challenges, while faster delivery rates (211 vs. 119 μg·min^−1^) reduce treatment time in distressed children, and near-complete drug utilization (>97% vs. 25–66%) ensures the dose predictability essential for weight-based pediatric regimens.

It should be noted that lower residual volume does not necessarily translate proportionally into increased pulmonary deposition, as downstream losses through the upper airways, breathing circuit and endotracheal tube may impose additional constraints on delivered lung dose.

### 4.5. Clinical Implications

Aerosol drug delivery of β2 agonists such as salbutamol is a vital initial therapeutic step in the management of the infant or child presenting to the emergency department (ED) with respiratory distress secondary to an acute airflow obstruction [44]. Many aerosol devices were historically designed for adults and larger children, with limited guidance or research on their adapted use in infants and younger children [55].

For patients that fail initial conventional treatments with aerosolized β2 agonists and oral or intravenous steroids, escalation of care to the critical care setting and non-invasive or invasive mechanical ventilation may be required. Effective drug delivery early in the clinical course is essential for successful therapy and perhaps the use of VMNs as the aerosol device of choice in the emergency department could minimize the drug dosage needed for treatment efficacy, reduce time to clinical improvement and decrease admission rates to critical care [37,53].

These findings are consistent with evidence from ED settings, where VMNs produced more rapid improvement in airflow than JNs in a randomized controlled trial in adults with severe asthma [56]. In pediatric emergency populations, VMNs resulted in faster clinical improvement, fewer treatments required and a lower probability of hospital admission compared with JNs [10,57,58].

VMN selection as the aerosol device of choice could have benefits extending from the emergency department to the critical care unit. Beyond clinical efficacy, the elimination of circuit interruptions for refilling preserves HFNT circuit integrity, while near-complete drug utilization reduces medication waste and pharmacy costs. These comparative performance data provide a timely evidence base for standardizing the highly variable nebulizer device selection and circuit configuration practices across pediatric critical care settings.

### 4.6. Limitations

There are several limitations to this study. The breathing profiles were designed to characterize a 9-month-old child and the findings should not be applied across all pediatric patients with potentially different breathing patterns, degrees of obstruction and patient size. The HFNT flow rates evaluated (2 and 11 L·min^−1^) reflect the operational range used in comparable published studies with this specific equipment and pediatric model [30,34,35]; however, weight-adjusted clinical recommendations for a 9-month-old infant may indicate flow rates of up to 16–24 L·min^−1^. The impact of higher flow rates on comparative device performance warrants further investigation. The consistent breathing patterns produced by the simulator do not represent the erratic patterns found in alert or distressed children, [59] and the model does not account for leakage or mask intolerance. Filter-based dose capture at the ETT slightly overestimates pulmonary delivery; however, this methodology is well-accepted, compliant with international standards and required by medical device regulators globally [29] and has shown good correlation with in vivo data [17,34,60]. The SAINT model, while anatomically correct, lacks airway cilia and a mucosal layer which possibly affects inhaled dose. Nebulizer position influences delivery during mechanical ventilation [41,42]. This study evaluated one specific JN (Aquineb) and one specific VMN (Aerogen Solo A-VMN). Findings should not be applied to all devices within either class. A single salbutamol formulation was used; performance with other formulations warrants separate investigation. Further studies are needed to establish superiority of delivery in the abnormal pediatric lung and to determine clinical impact in vivo.

## 5. Conclusions

This study demonstrates the importance of nebulizer device selection in both spontaneously breathing and mechanically ventilated pediatric patients, with the A-VMN delivering significantly increased quantity and rate of drug delivery and significantly reduced residual volumes post-nebulization compared to jet nebulizers. These findings could represent a dose–response effect if maintained in clinical settings. A-VMN use could improve drug delivery in the pediatric population, potentially reducing the need for back-to-back nebulizer treatments in spontaneously breathing patients and enhancing drug delivery efficiency in mechanically ventilated children, ultimately contributing to improved clinical outcomes. Further studies are needed to establish delivery superiority in pediatric patients with obstructive airway disease under clinically relevant conditions. This study provides a foundation for future clinical trials comparing nebulizer performance in vivo and determining the impact of device selection on clinical outcomes in pediatric respiratory care.

## Figures and Tables

**Table 1:** Pharmacopeial breath patterns for pediatric patients [27].

	Mechanical Ventilation	Spontaneous Breathing
**Breaths per minute**	25	25
**Tidal volume**	150	150
**I:E ratio**	1:2	1:2
**Positive end-expiratory pressure**	5 cm H_2_O	N/A
**F_i_O_2_**	0.21	0.21

**Table 2:** Salbutamol delivery for JN and VMN across delivery modalities.

	Drug Mass (μg)		Residual Volume (%)	
	JN	VMN	JN	VMN
**Mechanical ventilation**	307.84 ± 80.35	950.00 ± 63.83	75.18 ± 6.97	2.41 ± 1.08
**Spontaneous breathing**	388.24 ± 35.66	762.75 ± 187.32	76.73 ± 2.95	5.34 ± 1.56
**Blow-by**	13.73 ± 8.99	82.35 ± 28.06	75.12 ± 3.60	4.68 ± 0.61

## Data Availability

The original contributions presented in this study are included in the article. Further inquiries can be directed to the corresponding author.
